# Are implant-based treatments considered viable for patients with focal or florid cemento-osseous dysplasia? A systematic review

**DOI:** 10.1186/s40902-024-00432-x

**Published:** 2024-06-20

**Authors:** Setareh Hosseinpour, Mohammad Hadi Khademi, Maryam Erfani, Seyed Ali Mosaddad, Artak Heboyan

**Affiliations:** 1 Student Research Committee, School of Dentistry, Shiraz University of Medical Sciences, Shiraz, Iran; 2 Department of Research Analytics, Saveetha Dental College and Hospitals, Saveetha Institute of Medical and Technical Sciences, Saveetha University, Chennai, India; 3 Department of Prosthodontics, Faculty of Stomatology, Yerevan State Medical University after Mkhitar Heratsi, Yerevan, Armenia; 4 Department of Prosthodontics, School of Dentistry, Tehran University of Medical Sciences, Tehran, Iran

**Keywords:** Dental implants, Implant‐supported dental prosthesis, Survival rate, Florid cemento-osseous dysplasia, Fibrous dysplasia of bone

## Abstract

**Background:**

Focal and florid cemento-osseous dysplasia are benign fibro-osseous lesions affecting the quality and quantity of the jawbones. This study aimed to determine the viability of implant-based approaches in the affected patients.

**Main text:**

Different scientific databases, including PubMed/MEDLINE, Scopus, Web of Science, Embase, the Cochrane Library, and Google Scholar, were searched until October 8, 2023, using a pre-determined search strategy. Two reviewers screened the retrieved reports and extracted the required information from the included studies. The eligibility criteria included English-language case reports/series or clinical trials. The JBI critical appraisal checklist for case reports was used to assess the methodological quality of the included studies. Three studies were deemed eligible to be included in this study out of the initial 202 records found. Five implants were placed in three patients, positioned in the proximity of the lesion area, without any additional treatment to remove the pathology. The mandibular posterior area was the affected site in all patients. Only one implant failed in one patient after 16 years, which was attributed to peri-implantitis and not the lesion. Other implants demonstrated successful maintenance over follow-up periods.

**Conclusions:**

Although the number of the included records was relatively low to draw firm conclusions, it seems that implant-based treatments in patients with focal/florid cemento-osseous dysplasia could be viable, considering a conservative and well-planned approach.

**Supplementary Information:**

The online version contains supplementary material available at 10.1186/s40902-024-00432-x.

## Background

One of the most effective therapeutic strategies in dental procedures is oral rehabilitation using dental implants, which offer good esthetic and functional outcomes [1]. The quantity and quality of the bone tissue needed to meet primary implant stability, however, directly affects the outcome of this treatment [2, 3]. When determining the need for implant placement, it is essential to consider systematic and local risk factors impacting metabolism and bone remodeling [4]. Core conditions to consider in this assessment include osteoporosis, endocrine diseases, and primary bone pathologies, including dysplasia, cysts, and tumors [4, 5]. Dental implants are a complicated treatment option for those with dysplastic bone diseases such as fibro-osseous dysplasia. Bone structural alterations that affect its regular blood supply and plasticity are frequently linked to bone dysplasia’s inherent character and biological behavior, which may further complicate the osseointegration [6].

Cemento-osseous dysplasia (COD) is a set of fibro-osseous jawbone lesions with diverse clinical subtypes that can occur in various sites and at variable dimensions [7, 8]. Healthy bone replacement by fibrous/cementoid tissue is a hallmark of COD [9]. Three different types of COD can be distinguished by the site and size of the lesion: periapical COD (PCOD), which is restricted to the apex of a few adjacent mandibular anterior teeth; focal COD (FoCOD), which is limited to the apex of a single posterior tooth; and florid COD (FlCOD), which is more extensive and involves the jaws on multiple levels. Though its specific etiopathology is yet unknown, periodontal ligament reactive or dysplastic alterations are frequently thought to cause COD [10, 11]. Middle-aged African American females are more likely to develop COD, particularly in the mandible [12, 13]. Clinically, COD is typically asymptomatic and is frequently diagnosed incidentally by radiological examinations [8, 12, 13]. These pathologies are usually fixed in size; however, they can potentially expand far from the normal remodeling process and bone turnover rate, potentially leading to a noticeable enlargement of the alveolar process and resorption of the corresponding cortices. Patients may infrequently experience an appearance of swelling or a low-grade, fluctuating, poorly defined pain [14]. Osteomyelitis-like symptoms, including suppurative fistulas and mucosal lesions associated with pain, can develop if FlCODs are subsequently infected [14]. A radiolucent lesion, a mixed radiolucency-radiopacity lesion, and a radiopaque lesion with a radiolucent rim are the radiographic appearances of COD in its different phases of development [15]. Clinical and radiographic features may be sufficient to diagnose a COD lesion without histopathologic validations [16].

Only routine follow-up exams are advised for COD, and therapy is frequently unnecessary [17]. Still, there is uncertainty about the feasibility of implant-based treatments in COD patients. In COD lesions, the normal bone structure is replaced by fibroblasts and collagen fibers that include varying amounts of mineralized substances [18], resulting in a weakened bone matrix with underdeveloped stromal vasculature, which places the afflicted bone at risk for subsequent infections [19]. When dysplastic bone lesions in COD develop strong mineralization, poor vascularization and a high propensity for forming isolated bone cavities could complicate bone-based treatments like dental implantology. According to these findings, it may be necessary to reconsider some ideas regarding implant rehabilitation in dysplastic bone. Therefore, due to the growing rate of implant-based treatments in society, it is essential to evaluate the success of dental implants in such patients. To the authors’ knowledge, no previous systematic review study has focused on this topic. This study was thus aimed to systematically review the literature to evaluate the viability of dental implant placement in patients with FoCOD/FlCOD.

## Methods

### Study design and main research question

The Cochrane Handbook for Systematic Reviews and Preferred Reporting Items for Systematic Reviews and Meta-Analyses (PRISMA) were used to conduct this investigation [20]. The review protocol was registered at the Open Science Framework (10.17605/OSF.IO/TSHKJ). The main research question posed for this study was, “Could florid/focal cemento-osseous dysplasia affect the success rate of implant-based treatments in the corresponding patients?”.

### Search strategy

An electronic search was carried out in five electronic databases, including MEDLINE through PubMed, Embase, Scopus, Web of Science, and the Cochrane Library, up to October 8, 2023, using a pre-determined search strategy (Supplemental Table S1). The search methodology was only applied to English-language articles without considering the research publication dates. The search strategy combined keywords with the regulated terms (MeSH and ENTREE) whenever it was practicable to provide the most delicate method of identifying probable records. The reference lists of the chosen publications and applicable preceding studies were perused to identify any potentially relevant studies. Additionally, a search in the online database Google Scholar was conducted. Records were inputted into a reference management application (Endnote 20; Clarivate Analytics) for filtering purposes. The evaluators' inter-rater consistency for the literature screening technique was determined using Cohen's Kappa coefficient. The frequency of exact agreements amongst reviewers was used to calculate the kappa value (к).

### Eligibility criteria

The inclusion criteria comprised case reports, case series, and clinical studies published in English and peer-reviewed journals that investigated whether FoCOD/FlCOD could affect the success of dental implants in the affected patients. The exclusion specifications included studies published in languages other than English, studies that investigated the effectiveness of implant-based approaches in patients with other types of fibro-osseous pathologies, such as periapical COD, and other categories of investigations, such as ex vivo, in vivo, narrative/systematic reviews, posters, book sections; expert views, analyses with inadequate or invalid data, letters to the editor, editorial and commentary reports, short communications, and studies that failed to fulfill the eligibility prerequisites. Additionally, studies that excised/treated the lesion in any form before the implant surgery or those that applied bone graft materials before/during the surgical procedure were excluded.

### Study selection and data extraction

Using the EndNote 20 program, two reviewers (S.H. and M.H.K.) independently reviewed all retrieved studies based on their titles and abstracts, eliminating duplicates and irrelevant articles. The same screeners then completed the screening process by cross-referencing the full texts of the remaining possibly eligible publications with the inclusion/exclusion criteria. The screeners examined a random sample of 10% of the papers up for screening to calibrate them before the evaluation process began. Conflicts that arose throughout the screening process were settled by the two reviewers agreeing or consulting a third author (S.A.M.).

The following information was independently collected by two reviewers (S.H. and M.E.) from the selected records using a standard Excel sheet: first author, publication year, country, patients' demographic data and medical background/examinations, type of the edentulism, planned implant treatment, antibiotic therapy, prostheses type/loading time, follow‐up duration, and study outcomes. A fourth author (A.H.) was consulted during the data extraction stage to settle potential disputes.

### Quality assessment

Two assessors (S.H. and M.H.K.) independently assessed the included studies’ methodological quality using the JBI critical appraisal checklist for case reports, available at https://jbi.global/critical-appraisal-tools [21].

## Results

### Study selection

**Fig. 1:**
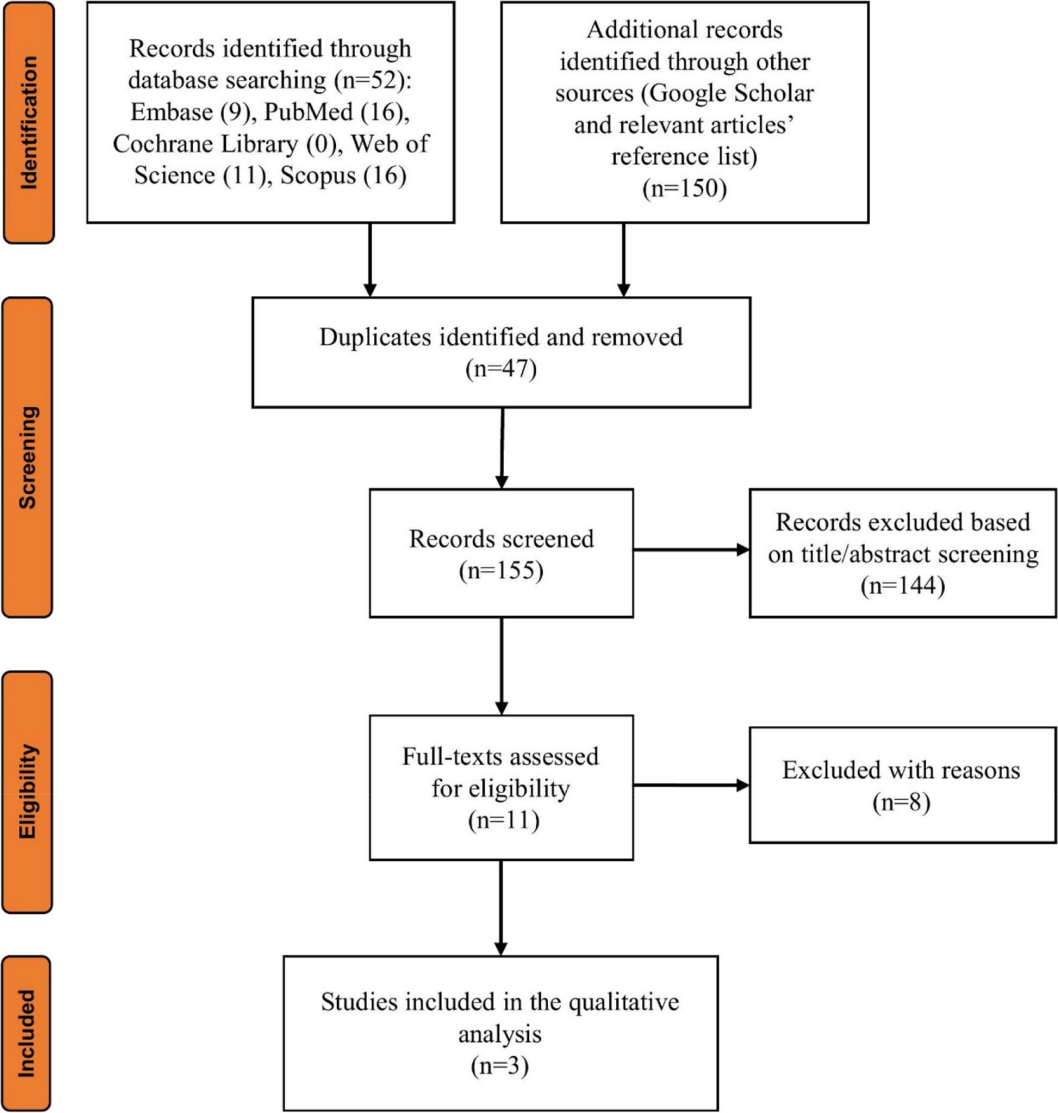
The PRISMA flowchart demonstrating the screening process results through different review stages

Out of the primary electronic database search, 202 records were identified. Duplicate reports (*n* = 47) and unrelated reports (*n* = 144) were discarded at the title/abstract assessment stage (к = 0.96). Eight entries were eliminated (к = 0.98) after the full texts of the remained articles (*n* = 11) had been reviewed: one study was only available as an abstract [22], one study was a short communication [23], the pathological lesion in one study [24] was mandibular fibrous dysplasia, in one study [10], the implants were placed in a normal anterior mandibular bone between the right mandibular area with a COD lesion and left mandibular area resected and reconstructed with plates and screws, one study [25] did not followed the placed implant and only discussed a surgical approach, in a study [26], in which the diagnosis of the lesion was made later at the time of explantation of a failed implant, there was no data regarding the presence of the lesion at the time of implant placement, in another study [27], the lesion was not diagnosed at the initial examinations and the intended implant region was grafted at the first surgery without any information regarding the excision or curettage of the undiagnosed radiolucent lesion, and the other one [28], first resected the lesion and the affected site surgically, then performed bone graft, and at a later stage the implants were placed in the grafted area, all contradicting the eligibility criteria designed for this study. Three publications [18, 29, 30] were ultimately chosen to form the basis of this systematic review. Cohen’s kappa coefficient values revealed perfect agreement between raters for both screening stages. Figure 1 illustrates the phased procedure of the screening.

### Study characteristics

The included studies were published between 2018 and 2020. Investigations were conducted in Iran [18], South Korea [29], and Palestine [30]. FoCOD and FlCOD were reported in one [29] and two studies [18, 30], respectively. Three patients with FoCOD or FlCOD were examined across the included studies. Based on the radiographical diagnosis, all implants were placed in/near a late-stage lesion. The selected studies’ characteristics are detailed in Table 1.

**Table 1:** Included studies’ characteristics focused on implant treatments in patients with FoCOD and FlCOD

Author/year/reference	Country	Lesion type/location/stage	Demographics (age/sex/sample size)	Medical history/examination	Edentulism	Implant(s) (number/location/surgical approach/dimensions/brand) placed in the lesion	Antibiotic therapy	The type of prosthesis fabricated/loading time	Follow-up duration	The final result of the implant treatment	Study outcome(s)
Esfahanizadeh and Yousefi 2018 [18]	Iran	FlCOD/mandibular molars at both sides/late-stage	62/F/one patient	No prior familial or systemic condition/ normal blood test results	Mandibular bilateral posterior partial edentulism	Two implants/mandibular left first and second molars (#36 and #37)/two-staged/not reported/not reported	Not reported	Fixed restorations/ after 6 months	2, 4, 6, 12, and 18 months	Successful and maintained until the latest reported follow-up	1. There were no changes in the size/features of the lesion at either of the follow-ups 2. The implants were well-osseointegrated and adequately functional
Park et al. 2019 [29]	South Korea	FoCOD/right posterior mandibular edentulous ridge/late-stage	39/M/one patient	Smoker, occasional alcohol use, no prior systemic condition/ no lab tests reported	Full maxillary and mandibular edentulism	One implant/ mandibular right first molar (#46)/two-staged/4 × 10 mm, external hex/3i T3, Zimmer Biomet	Cefadroxil, three 500 mg tablets/day postoperatively for 10 days	1. A provisional complete denture/after 1 week 2. Fixed prosthesis/after 6 months	First, at 6 months, then at 3, 6, 10, 14, 15, 16 years	Failed and explanted due to peri-implantitis after 16 years	1. At 6 months, the implant was well-osseointegrated 2. At 15 years: preliminary indications of bone loss surrounding the #45 implant, whereas the #46 implant appeared stable and showed no evident alterations 3. At 16 years: clinical signs of gingival edema, hemorrhage, pus discharge, and discomfort during mastication appeared as peri-implantitis 4. Although a high implant-to-tissue contact was achieved, the implant failed due to peri-implantitis
Shadid and Kujan 2020 [30]	Palestine	FlCOD/incisor, premolar, and molar areas of the mandibular right and left regions/late-stage	44/F/one patient	No prior systemic condition/no lab tests reported	Mandibular right posterior partial edentulism	Two implants/mandibular right first and second molars (#46 and #47)/two-staged/4.6 × 12 mm, internal hex/BioHorizons, Birmingham, AL, USA	Prophylaxis with 500 mg/125 mg amoxicillin/clavulanic acid 1 h before surgery	Fixed cement-retained metal-ceramic splinted restorations/ after 6 months	At 5 and 6 months, at 1 year, then biannually until 8 years	The absence of peri-implant diseases, including mucositis and peri-implantitis with bone loss up to an 8-year follow-up period, demonstrated an explicit functional integration between dysplastic bone and the loaded implant	1. In follow-ups: a distinct radiolucency emerged on the distal aspect of the #47 implant's apex, although neither the lesion's size nor peri-implant bone loss was noted in follow-ups 2. At 6 years: successful implant osseointegration and optimum function 3. At 8 years: maintained osseointegration with healthy peri-implant soft tissue and no significant marginal bone loss

*FoCOD* Focal cemento-osseous dysplasia, *FlCOD* Florid cemento-osseous dysplasia, *F* Female, *M* Male

### Patients’ characteristics, implant, and prosthodontic features

Two patients were female, ages 62 and 44, and one was male, age 39. The patients received five implants, all placed in mandibular posterior areas at the bone level through a two-staged approach. In one study, the antibiotic therapy was performed preoperatively [30], while in another one [29], it was prescribed postoperatively; the use of antibiotics was not reported in one study [18]. In all reports, the prosthetic phase for fabricating final restorations was conducted after 6 months; however, in one study [29], a provisional removable complete denture was given to the patient after 1 week. Prostheses were all fixed-type; the type of restoration in one study [30] was reported as a splinted two-unit cement-retained metal-ceramic restoration, while in the other two, it was not described. However, based on the figures provided, it could be speculated that the restorations in one study [18] were also splinted two-unit cement-retained metal-ceramic, and the other one [29] were screw-retained metal-ceramics.

### Follow-up and survival

The minimum follow-up time was 18 months [18], while the longest lasted 192 months [29]. Of the five implants placed, only one showed failure and was explanted. However, the authors [29] reported that the developed peri-implantitis was the main reason for the failure, which was verified by further histopathological examinations; a highly integrated implant-to-central bony section of the lesion was found; the implant failure occurred after 16 years of prosthetic loading. The studies provided no numeral reporting regarding radiographic marginal bone loss and probing depths measuring.

### Quality assessment

Table 2 presents the results of the quality assessment of the listed publications. Except for two studies [18, 30] where the patient history and the timeframe reported were unclear, all studies received complete scores according to the checklist used to grade publications. The likelihood of unforeseen complications or adverse events was not disclosed in the abovementioned reports [18, 30].

**Table 2:** Results and detailed scoring of the included studies’ quality assessment using the JBI critical appraisal checklist for case reports

Studies/assessment criteria	Q1	Q2	Q3	Q4	Q5	Q6	Q7	Q8
Esfahanizadeh and Yousefi	Y	U	Y	Y	Y	Y	N	Y
Park et al	Y	Y	Y	Y	Y	Y	Y	Y
Shadid and Kujan	Y	U	Y	Y	Y	Y	N	Y

*Q1* were patient’s demographic characteristics clearly described? *Q2* was the patient’s history clearly described and presented as a timeline? *Q3* was the current clinical condition of the patient on presentation clearly described? *Q4* were diagnostic tests or assessment methods and the results clearly described; *Q5* was the intervention(s) or treatment procedure(s) clearly described? *Q6* was the post-intervention clinical condition clearly described? *Q7* were adverse events (harms) or unanticipated events identified and described? *Q8* does the case report provide takeaway lessons? *Y* yes, *N* no, *U* unclear; *NA* not applicable

## Discussion

It has been clarified that in individuals with FCOD, extractions, and even elective surgical treatments should be avoided. Persistent bone resorption of the edentulous ridge over time, tooth/root extraction in the proximity of a COD lesion, or implant drilling could expose the cementum-like tissue (CLT), leading to complications like infection [31]. CLT, or cementum-like tissue, is the histological interpretation of the central sclerotic mass adhered to the implant surface. According to reports, the main issues include inadequate healing, sequestrum development, infection risk, and jaw fracture [32–34]. Following tooth extraction close to the COD lesion, Waldron et al. observed inadequate socket healing and sequestrum development [31]. A more severe consequence that has also been observed is osteomyelitis [13, 35]. The gradual deposition of CLT appears to raise the risk of osteomyelitis and subsequent infections [36, 37].

The bone at the osteotomy site might become necrosed due to the overheating of the surgical drills, especially in the case of inadequate cooling provided by copious irrigation [38]. If this induced inflammatory process spreads to the sclerotic bone within the lesion, necrosis could turn into osteomyelitis [39]. Furthermore, the direct exposure of the highly hypovascular tissue to the oral cavity may cause these adverse effects [35, 40, 41]. The avascular character of advanced FCOD lesions may make it more difficult for the implants to integrate into the bone. If a secondary infection does arise, it will likely be aggressive and challenging to treat. Poor healing, an increased risk of infection, and a fractured jaw have been identified as the main anticipated complications [32–34]. Because of the potential for these effects to adversely affect the long-term durability of dental implants in FCOD lesions, Sukegawa et al. suggested removing these lesions before placing implants [40]. Therefore, a strict infection control protocol at the time of surgery, achieving an ideal peri-implant soft tissue integration/thickness throughout the healing phase, a well-contoured emergence profile and prosthetic restoration(s), regular maintenance, and adhering to appropriate oral hygiene practiced by the patients are essential elements in planning an implant-based treatment to isolate and conserve the central sclerotic mass and the fibrotic rim surrounding it.

Additionally, if implant therapy is indicated, it ought to be inserted only into a late-stage FCOD lesion with a heavily calcified CLT due to the young tissue present [29]. Implant failures in COD lesions have also been linked to the immaturity of the COD content [26, 27]. Implant placement into COD lesions of an early or intermediate stage may result in failed osseointegration due to restricted BIC. Conversely, it has been demonstrated that late-stage COD lesions have a higher percentage of CLT than early- and intermediate-stage lesions [36, 37] because lesion maturation is characterized by the gradual deposition of components resembling cementum [13]. The advanced lesions persist in growing, combining, and undergoing additional significant radiopacification [13, 42]. In the study by Park et al. [29], although the implant failed after 16 years, micro-CT and histopathological evaluations demonstrated a high integration of the implant to the surrounding bony mass. The specimen's micro-CT data showed that, with a very high bone mineral density, no visible trabecular pattern, and no gap at the implant-lesion interface, the integration of the implant into the sclerotic mass mirrored the typical osseointegration process observed through implant placement in a healthy bone with an extremely high ratio of tissue-to-implant contact, comparable to BIC. They noticed marginal bone loss induced by peri-implantitis at the crestal area close to the implant platform during the 16-year follow-up session. Bone loss was progressive due to peri-implantitis affecting the loose connective tissues around the FCOD lesion. Therefore, this enhanced CLT-to-implant contact reported by Park et al. [29] provides a proper explanation for the increased likelihood of effective integration of an implant placed in the late-stage lesion. Additionally, implant placement should only be considered once the surrounding inflammation and endodontic or periodontal diseases have been adequately treated. Establishing a well-managed maintenance program is also advisable to keep potentially infectious sources away from the gingiva, implants, and neighboring teeth [29].

To the authors' knowledge, this study is the first systematic review of the practicality of dental implants as a treatment option in patients with FoCOD/FlCOD. There have, however, been few studies—mostly case reports—that have provided data on the targeted subject. In numerous instances, such as assessing soft and hard tissues over time, the results were summed together rather than described as patient-specific. The small number of patients recruited and implants placed, together with the ambiguity surrounding the methodology for implant placement and the prosthetic approaches used, limited the variety of data for investigation. There might be some missing data because only studies published in English had been considered.

## Conclusions

There were not sufficient studies included to allow for the formulation of strong conclusions. However, all limitations considered, implant rehabilitation of the edentulous area adjacent to COD lesions might be regarded as a feasible option in late-stage conditions, provided that stringent infection control protocol is followed, and a minimally invasive technique is used. The reliability of dental implant rehabilitation in the long- or even short-term for patients with cementosseous dysplasia is not well-established. Therefore, routine clinical and radiological follow-ups are necessary, and patients must maintain good oral hygiene and routinely attend follow-up appointments.

### Supplementary Information

Supplementary Material 1: Supplemental Table S1. Different search strategies and the corresponding keywords used in searching scientific databases.

## Data Availability

The data presented in this study are available upon request from the corresponding author.

## Abbreviations

COD: Cemento-osseous dysplasia
PCOD: Periapical cemento-osseous dysplasia
FoCOD: Focal cemento-osseous dysplasia
FlCOD: Florid cemento-osseous dysplasia
CLT: Cementum-like tissue
