# Supplementary material for: Are implant-based treatments considered viable for patients with focal or florid cemento-osseous dysplasia? A systematic review
Source: Maxillofac Plast Reconstr Surg. 2024 Jun 20;46(1):23. doi: 10.1186/s40902-024-00432-x (PMC11189879; doi:10.1186/s40902-024-00432-x)
Supplement: Supplementary file 1 — Supplementary Material 1: Supplemental Table S1. Different search strategies and the corresponding keywords used in searching scientific databases. [file 40902_2024_432_MOESM1_ESM.docx]

**Supplemental Table 1.** Different search strategies and the corresponding keywords used in searching scientific databases.

| **Database** | **Keywords/Search strategy** |
| --- | --- |
| **Pubmed/Medline** | ("Dental Implants"[Mesh Terms] OR "Dental Implants"[Title/Abstract] OR "Dental Implant"[Title/Abstract] OR "Mini Dental Implant"[Title/Abstract] OR "Mini Dental Implants"[Title/Abstract] OR "Surgical Dental Prostheses"[Title/Abstract] OR "Surgical Dental Prosthesis"[Title/Abstract] OR "Dental Implants, Single-Tooth"[Mesh Terms] OR "Single-Tooth Implants"[Title/Abstract] OR "Single Tooth Implants"[Title/Abstract] OR "Single-Tooth Implant"[Title/Abstract] OR "Single-Tooth Dental Implants"[Title/Abstract] OR "Single Tooth Dental Implants"[Title/Abstract] OR "Single-Tooth Dental Implant"[Title/Abstract] OR "Single Tooth Dental Implant"[Title/Abstract] OR "Dental Implant-Abutment Design"[Mesh Terms] OR "Dental Implant-Abutment Design"[Title/Abstract] OR "Dental Implant Abutment Design"[Title/Abstract] OR "Dental Implant-Abutment Designs"[Title/Abstract] OR "Dental Implant Abutment Designs"[Title/Abstract] OR "Dental Implant-Abutment Interface"[Title/Abstract] OR "Dental Implant Abutment Interface"[Title/Abstract] OR "Dental Implant-Abutment Interfaces"[Title/Abstract] OR "Dental Implant-Abutment Connection"[Title/Abstract] OR "Dental Implant Abutment Connection"[Title/Abstract] OR "Dental Implant-Abutment Connections"[Title/Abstract] OR "Dental Implantation"[Mesh Terms] OR "Dental Implantation"[Title/Abstract] OR "Dental Implant Therapy"[Title/Abstract] OR "Dental Implant Therapies"[Title/Abstract] OR "Dental Prosthesis Implantation"[Title/Abstract] OR "Dental Prosthesis Implantations"[Title/Abstract] OR "Dental Implantation, Endosseous"[Mesh Terms] OR "Endosseous Dental Implant Therapy"[Title/Abstract] OR "Endosseous Dental Implantation"[Title/Abstract] OR "Osseointegrated Dental Implantation"[Title/Abstract] OR "Endosseous Implantation"[Title/Abstract]) AND ("Florid cemento-osseous dysplasia"[Supplementary Concept] OR "Florid cemento-osseous dysplasia"[Title/Abstract] OR "florid cemento osseous dysplasia"[Title/Abstract] OR "florid osseous dysplasia"[Title/Abstract] OR "florid ossifying dysplasia"[Title/Abstract] OR "Florid cemento-ossifying dysplasia"[Title/Abstract] OR "cemento-osseous dysplasia"[Title/Abstract] OR "cemento-ossifying dysplasia"[Title/Abstract] OR "Focal cemento-osseous dysplasia"[Title/Abstract] OR "focal cemento osseous dysplasia"[Title/Abstract] OR "focal osseous dysplasia"[Title/Abstract] OR "focal ossifying dysplasia"[Title/Abstract] OR "focal cemento-ossifying dysplasia"[Title/Abstract] OR Cementoma[Mesh Terms] OR Cementoma[Title/Abstract] OR Cementomas[Title/Abstract] OR "Cemento-Ossifying Fibroma"[Title/Abstract] OR "Cemento Ossifying Fibroma"[Title/Abstract] OR "Cementifying Fibroma of Maxilla"[Title/Abstract] OR "Maxilla Cementifying Fibroma"[Title/Abstract] OR "Maxilla Cementifying Fibromas"[Title/Abstract] OR "Ossifying Fibroma of Maxilla"[Title/Abstract] OR "Maxilla Ossifying Fibroma"[Title/Abstract] OR "Maxilla Ossifying Fibromas"[Title/Abstract] OR "Maxillary Cementifying Fibroma"[Title/Abstract] OR "Maxillary Cementifying Fibromas"[Title/Abstract] OR "Cementifying Fibroma of Mandible"[Title/Abstract] OR "Mandible Cementifying Fibroma"[Title/Abstract] OR "Mandible Cementifying Fibromas"[Title/Abstract] OR "Ossifying Fibroma of Mandible"[Title/Abstract] OR "Mandible Ossifying Fibroma"[Title/Abstract] OR "Mandible Ossifying Fibromas"[Title/Abstract] OR "Mandibular Cementifying Fibroma"[Title/Abstract] OR "Mandibular Cementifying Fibromas"[Title/Abstract]) |
| **Scopus** | TITLE-ABS-KEY("Dental Implants" OR "Dental Implant" OR "Mini Dental Implant" OR "Mini Dental Implants" OR "Surgical Dental Prostheses" OR "Surgical Dental Prosthesis" OR "Dental Implants, Single-Tooth" OR "Single-Tooth Implants" OR "Single Tooth Implants" OR "Single-Tooth Implant" OR "Single-Tooth Dental Implants" OR "Single Tooth Dental Implants" OR "Single-Tooth Dental Implant" OR "Single Tooth Dental Implant" OR "Dental Implant-Abutment Design" OR "Dental Implant Abutment Design" OR "Dental Implant-Abutment Designs" OR "Dental Implant Abutment Designs" OR "Dental Implant-Abutment Interface" OR "Dental Implant Abutment Interface" OR "Dental Implant-Abutment Interfaces" OR "Dental Implant-Abutment Connection" OR "Dental Implant Abutment Connection" OR "Dental Implant-Abutment Connections" OR "Dental Implantation" OR "Dental Implant Therapy" OR "Dental Implant Therapies" OR "Dental Prosthesis Implantation" OR "Dental Prosthesis Implantations" OR "Dental Implantation, Endosseous" OR "Endosseous Dental Implant Therapy" OR "Endosseous Dental Implantation" OR "Osseointegrated Dental Implantation" OR "Endosseous Implantation") AND TITLE-ABS-KEY("Florid cemento-osseous dysplasia" OR "florid cemento osseous dysplasia" OR "florid osseous dysplasia" OR "florid ossifying dysplasia" OR "Florid cemento-ossifying dysplasia" OR "cemento-osseous dysplasia" OR "cemento-ossifying dysplasia" OR "Focal cemento-osseous dysplasia" OR "focal cemento osseous dysplasia" OR "focal osseous dysplasia" OR "focal ossifying dysplasia" OR "focal cemento-ossifying dysplasia" OR Cementoma OR Cementomas OR "Cemento-Ossifying Fibroma" OR "Cemento Ossifying Fibroma" OR "Cementifying Fibroma of Maxilla" OR "Maxilla Cementifying Fibroma" OR "Maxilla Cementifying Fibromas" OR "Ossifying Fibroma of Maxilla" OR "Maxilla Ossifying Fibroma" OR "Maxilla Ossifying Fibromas" OR "Maxillary Cementifying Fibroma" OR "Maxillary Cementifying Fibromas" OR "Cementifying Fibroma of Mandible" OR "Mandible Cementifying Fibroma" OR "Mandible Cementifying Fibromas" OR "Ossifying Fibroma of Mandible" OR "Mandible Ossifying Fibroma" OR "Mandible Ossifying Fibromas" OR "Mandibular Cementifying Fibroma" OR "Mandibular Cementifying Fibromas") |
| **Embase** | ('Dental Implants':ti,ab,kw OR 'Dental Implant':ti,ab,kw OR 'Mini Dental Implant':ti,ab,kw OR 'Mini Dental Implants':ti,ab,kw OR 'Surgical Dental Prostheses':ti,ab,kw OR 'Surgical Dental Prosthesis':ti,ab,kw OR 'Dental Implants, Single-Tooth':ti,ab,kw OR 'Single-Tooth Implants':ti,ab,kw OR 'Single Tooth Implants':ti,ab,kw OR 'Single-Tooth Implant':ti,ab,kw OR 'Single-Tooth Dental Implants':ti,ab,kw OR 'Single Tooth Dental Implants':ti,ab,kw OR 'Single-Tooth Dental Implant':ti,ab,kw OR 'Single Tooth Dental Implant':ti,ab,kw OR 'Dental Implant-Abutment Design':ti,ab,kw OR 'Dental Implant Abutment Design':ti,ab,kw OR 'Dental Implant-Abutment Designs':ti,ab,kw OR 'Dental Implant Abutment Designs':ti,ab,kw OR 'Dental Implant-Abutment Interface':ti,ab,kw OR 'Dental Implant Abutment Interface':ti,ab,kw OR 'Dental Implant-Abutment Interfaces':ti,ab,kw OR 'Dental Implant-Abutment Connection':ti,ab,kw OR 'Dental Implant Abutment Connection':ti,ab,kw OR 'Dental Implant-Abutment Connections':ti,ab,kw OR 'Dental Implantation':ti,ab,kw OR 'Dental Implant Therapy':ti,ab,kw OR 'Dental Implant Therapies':ti,ab,kw OR 'Dental Prosthesis Implantation':ti,ab,kw OR 'Dental Prosthesis Implantations':ti,ab,kw OR 'Dental Implantation, Endosseous':ti,ab,kw OR 'Endosseous Dental Implant Therapy':ti,ab,kw OR 'Endosseous Dental Implantation':ti,ab,kw OR 'Osseointegrated Dental Implantation':ti,ab,kw OR 'Endosseous Implantation':ti,ab,kw) AND ('Florid cemento-osseous dysplasia':ti,ab,kw OR 'florid cemento osseous dysplasia':ti,ab,kw OR 'florid osseous dysplasia':ti,ab,kw OR 'florid ossifying dysplasia':ti,ab,kw OR 'Florid cemento-ossifying dysplasia':ti,ab,kw OR 'cemento-osseous dysplasia':ti,ab,kw OR 'cemento-ossifying dysplasia':ti,ab,kw OR 'Focal cemento-osseous dysplasia':ti,ab,kw OR 'focal cemento osseous dysplasia':ti,ab,kw OR 'focal osseous dysplasia':ti,ab,kw OR 'focal ossifying dysplasia':ti,ab,kw OR 'focal cemento-ossifying dysplasia':ti,ab,kw OR Cementoma:ti,ab,kw OR Cementomas:ti,ab,kw OR 'Cemento-Ossifying Fibroma':ti,ab,kw OR 'Cemento Ossifying Fibroma':ti,ab,kw OR 'Cementifying Fibroma of Maxilla':ti,ab,kw OR 'Maxilla Cementifying Fibroma':ti,ab,kw OR 'Maxilla Cementifying Fibromas':ti,ab,kw OR 'Ossifying Fibroma of Maxilla':ti,ab,kw OR 'Maxilla Ossifying Fibroma':ti,ab,kw OR 'Maxilla Ossifying Fibromas':ti,ab,kw OR 'Maxillary Cementifying Fibroma':ti,ab,kw OR 'Maxillary Cementifying Fibromas':ti,ab,kw OR 'Cementifying Fibroma of Mandible':ti,ab,kw OR 'Mandible Cementifying Fibroma':ti,ab,kw OR 'Mandible Cementifying Fibromas':ti,ab,kw OR 'Ossifying Fibroma of Mandible' :ti,ab,kw OR 'Mandible Ossifying Fibroma':ti,ab,kw OR 'Mandible Ossifying Fibromas':ti,ab,kw OR 'Mandibular Cementifying Fibroma':ti,ab,kw OR 'Mandibular Cementifying Fibromas':ti,ab,kw) |
| **Web of Science** | TS=("Dental Implants" OR "Dental Implant" OR "Mini Dental Implant" OR "Mini Dental Implants" OR "Surgical Dental Prostheses" OR "Surgical Dental Prosthesis" OR "Dental Implants, Single-Tooth" OR "Single-Tooth Implants" OR "Single Tooth Implants" OR "Single-Tooth Implant" OR "Single-Tooth Dental Implants" OR "Single Tooth Dental Implants" OR "Single-Tooth Dental Implant" OR "Single Tooth Dental Implant" OR "Dental Implant-Abutment Design" OR "Dental Implant Abutment Design" OR "Dental Implant-Abutment Designs" OR "Dental Implant Abutment Designs" OR "Dental Implant-Abutment Interface" OR "Dental Implant Abutment Interface" OR "Dental Implant-Abutment Interfaces" OR "Dental Implant-Abutment Connection" OR "Dental Implant Abutment Connection" OR "Dental Implant-Abutment Connections" OR "Dental Implantation" OR "Dental Implant Therapy" OR "Dental Implant Therapies" OR "Dental Prosthesis Implantation" OR "Dental Prosthesis Implantations" OR "Dental Implantation, Endosseous" OR "Endosseous Dental Implant Therapy" OR "Endosseous Dental Implantation" OR "Osseointegrated Dental Implantation" OR "Endosseous Implantation") AND TS=("Florid cemento-osseous dysplasia" OR "florid cemento osseous dysplasia" OR "florid osseous dysplasia" OR "florid ossifying dysplasia" OR "Florid cemento-ossifying dysplasia" OR "cemento-osseous dysplasia" OR "cemento-ossifying dysplasia" OR "Focal cemento-osseous dysplasia" OR "focal cemento osseous dysplasia" OR "focal osseous dysplasia" OR "focal ossifying dysplasia" OR "focal cemento-ossifying dysplasia" OR Cementoma OR Cementomas OR "Cemento-Ossifying Fibroma" OR "Cemento Ossifying Fibroma" OR "Cementifying Fibroma of Maxilla" OR "Maxilla Cementifying Fibroma" OR "Maxilla Cementifying Fibromas" OR "Ossifying Fibroma of Maxilla" OR "Maxilla Ossifying Fibroma" OR "Maxilla Ossifying Fibromas" OR "Maxillary Cementifying Fibroma" OR "Maxillary Cementifying Fibromas" OR "Cementifying Fibroma of Mandible" OR "Mandible Cementifying Fibroma" OR "Mandible Cementifying Fibromas" OR "Ossifying Fibroma of Mandible" OR "Mandible Ossifying Fibroma" OR "Mandible Ossifying Fibromas" OR "Mandibular Cementifying Fibroma" OR "Mandibular Cementifying Fibromas") |
| **Google Scholar** | ("Dental Implants" OR "Dental Implant" OR "Dental Implantation") AND ("Florid cemento-osseous dysplasia" OR "Focal cemento-osseous dysplasia" OR Cementoma) |
| **The Cochrane library** | \| #1 \| MeSH descriptor: [Dental Implants] explode all trees \| 2083 \| \| --- \| --- \| --- \| \| #2 \| MeSH descriptor: [Dental Implants, Single-Tooth] explode all trees \| 409 \| \| #3 \| MeSH descriptor: [Dental Implant-Abutment Design] explode all trees \| 135 \| \| #4 \| MeSH descriptor: [Dental Implantation] explode all trees \| 1627 \| \| #5 \| MeSH descriptor: [Dental Implantation, Endosseous] explode all trees \| 1578 \| \| #6 \| MeSH descriptor: [Cementoma] explode all trees \| 1 \| \| #7 \| ("Dental Implants" OR "Dental Implant" OR "Mini Dental Implant" OR "Mini Dental Implants" OR "Surgical Dental Prostheses" OR "Surgical Dental Prosthesis" OR "Single-Tooth Implants" OR "Single Tooth Implants" OR "Single-Tooth Implant" OR "Single-Tooth Dental Implants" OR "Single Tooth Dental Implants" OR "Single-Tooth Dental Implant" OR "Single Tooth Dental Implant" OR "Dental Implant-Abutment Design" OR "Dental Implant Abutment Design" OR "Dental Implant-Abutment Designs" OR "Dental Implant Abutment Designs" OR "Dental Implant-Abutment Interface" OR "Dental Implant Abutment Interface" OR "Dental Implant-Abutment Interfaces" OR "Dental Implant-Abutment Connection" OR "Dental Implant Abutment Connection" OR "Dental Implant-Abutment Connections" OR "Dental Implantation" OR "Dental Implant Therapy" OR "Dental Implant Therapies" OR "Dental Prosthesis Implantation" OR "Dental Prosthesis Implantations" OR "Endosseous Dental Implant Therapy" OR "Endosseous Dental Implantation" OR "Osseointegrated Dental Implantation" OR "Endosseous Implantation"):ti,ab,kw \| 4079 \| \| #8 \| ("Florid cemento-osseous dysplasia" OR "florid cemento osseous dysplasia" OR "florid osseous dysplasia" OR "florid ossifying dysplasia" OR "Florid cemento-ossifying dysplasia" OR "cemento-osseous dysplasia" OR "cemento-ossifying dysplasia" OR"Focal cemento-osseous dysplasia" OR "focal cemento osseous dysplasia" OR "focal osseous dysplasia" OR "focal ossifying dysplasia" OR "focal cemento-ossifying dysplasia" OR Cementoma OR Cementomas OR "Cemento-Ossifying Fibroma" OR "Cemento Ossifying Fibroma" OR "Cementifying Fibroma of Maxilla" OR "Maxilla Cementifying Fibroma" OR "Maxilla Cementifying Fibromas" OR "Ossifying Fibroma of Maxilla" OR "Maxilla Ossifying Fibroma" OR "Maxilla Ossifying Fibromas" OR "Maxillary Cementifying Fibroma" OR "Maxillary Cementifying Fibromas" OR "Cementifying Fibroma of Mandible" OR "Mandible Cementifying Fibroma" OR "Mandible Cementifying Fibromas" OR "Ossifying Fibroma of Mandible" OR "Mandible Ossifying Fibroma" OR "Mandible Ossifying Fibromas" OR "Mandibular Cementifying Fibroma" OR "Mandibular Cementifying Fibromas"):ti,ab,kw \| 1 \| \| #9 \| (#1 OR #2 OR #3 OR #4 OR #5 OR #7 ) AND (#8) \| 0 \| |
